# Extraction of Ejection Fraction from Echocardiography Notes for Constructing a Cohort of Patients having Heart Failure with reduced Ejection Fraction (HFrEF)

**DOI:** 10.1007/s10916-018-1066-7

**Published:** 2018-09-25

**Authors:** Kavishwar B. Wagholikar, Christina M. Fischer, Alyssa Goodson, Christopher D. Herrick, Martin Rees, Eloy Toscano, Calum A. MacRae, Benjamin M. Scirica, Akshay S. Desai, Shawn N. Murphy

**Affiliations:** 1 Harvard Medical School, Boston, MA USA; 2 Massachusetts General Hospital, Boston, MA USA; 3 Partners Healthcare, Boston, MA USA; 4 Brigham Women’s Hospital, Boston, MA USA

**Keywords:** Ejection fraction, Natural language processing, Regular expression, Cardiology, Echocardiogram

## Abstract

Left ventricular ejection fraction (LVEF) is an important prognostic indicator of cardiovascular outcomes. It is used clinically to determine the indication for several therapeutic interventions. LVEF is most commonly derived using in-line tools and some manual assessment by cardiologists from standardized echocardiographic views. LVEF is typically documented in free-text reports, and variation in LVEF documentation pose a challenge for the extraction and utilization of LVEF in computer-based clinical workflows. To address this problem, we developed a computerized algorithm to extract LVEF from echocardiography reports for the identification of patients having heart failure with reduced ejection fraction (HFrEF) for therapeutic intervention at a large healthcare system. We processed echocardiogram reports for 57,158 patients with coded diagnosis of Heart Failure that visited the healthcare system over a two-year period. Our algorithm identified a total of 3910 patients with reduced ejection fraction. Of the 46,634 echocardiography reports processed, 97% included a mention of LVEF. Of these reports, 85% contained numerical ejection fraction values, 9% contained ranges, and the remaining 6% contained qualitative descriptions. Overall, 18% of extracted numerical LVEFs were ≤ 40%. Furthermore, manual validation for a sample of 339 reports yielded an accuracy of 1.0. Our study demonstrates that a regular expression-based approach can accurately extract LVEF from echocardiograms, and is useful for delineating heart-failure patients with reduced ejection fraction.

## Introduction

Left ventricular ejection fraction (LVEF) is an important prognostic indicator of cardiovascular outcomes [1, 2]. Clinically, it is used to guide the therapeutic pathways for patients having heart failure [3]. LVEF is estimated manually by cardiologists during echocardiographic examinations, and is typically documented in free-text reports [4]. Variation in LVEF documentation from the echocardiograms pose a challenge for the extraction and utilization of LVEF in computer-based clinical workflows.

## Significance of left ventricular ejection fraction (LVEF)

During each heartbeat, when the heart contracts, it ejects blood from the two pumping chambers called ventricles, and the ventricles refill with blood when the heart relaxes. The term “ejection fraction” (EF) refers to the percentage of blood that is pumped out of a filled ventricle with each heartbeat. A LVEF of 55% or higher is considered normal under physiologic loading conditions, with an EF of 50% or lower being considered reduced. Notably, EFs between 50 and 55% are considered “borderline” [5, 6].

Several imaging modalities can be used to measure LVEF, including echocardiography, magnetic resonance imaging (MRI), computed tomography (CT), radionuclide angiography, and gated myocardial perfusion single-photon emission computed tomography (GSPECT) [4]. Currently, MRI is widely considered the gold standard for EF measurement, largely due to the utility of tomographic techniques in overcoming the need for radiation exposure and dealing with the geometric complexity of the cardiac chambers, particularly of the right ventricle [4, 7, 8].

EF on echocardiography is typically measured only in the left ventricle (LV), as the left ventricle is fully accessible using standard echocardiographic views and the geometry of the chamber allows robust estimates of EF as a parameter from orthogonal 2D images using the prolate ellipse as a model. While these estimates have been shown to be reliable, there remains a degree of operator dependence in most echocardiographic assessments of EF. Nevertheless, echocardiog-raphically estimated EF remains the most widely used approach, due to its low cost, absence of radiation and increasingly wide availability.

## Therapeutic pathways for patients with reduced ejection fraction

The management of patients with reduced EF, whether they have the symptoms of heart failure or not, has been an area of major advance in the last two decades. Randomized control trials have identified multiple drug classes which reduce mortality and morbidity in those with HF and reduced EF (HFrEF). Several subsets of HFrEF are also known to benefit from the implantation of automated implantable cardiodefi-brillators. Similarly, trials combining EF with electrical information have identified subsets of patients who benefit from very specific forms of cardiac electrical resynchronization through implanted pacemakers. Together, these advances have firmly anchored the modern management of heart failure around echocardiographic data.

## Challenge for LVEF extraction

Ejection fraction (EF) is often reported in free-text format, and its extraction remains challenging due to considerable variation in the manner of documentation in echocardiography reports. EF is reported in different areas of reports, or in different formats, either as a number range or as qualitative descriptions (see Table 1).

**Table:** 

Type of Mention	Example Excerpts
Number	The left ventricular ejection fraction is 60% Ejection Fraction 20% (A) (Range: 50 - 75) lv ejection fraction 66%
Range	Estimated left ventricular ejection fraction is 45-50% The LVEF is visually estimated at 30-35% LVEF by visual estimation is around 35%
Qualitative	LVEF appears at the lower limits of normal Left ventricular systolic function is moderately impaired Left ventricular systolic function is moderately decreased

**Table 1:** Distribution of high level echocardiogram categorization of based on mentions of anchor terms for ejection fraction

Group Description	Count	%
EF in tabular pattern	34,716	74
No section of left ventricle	1127	2
Left ventricle section	9358	20
No keywords for ejection fraction	1433	3
Total	46,634	100

## Previous research on extracting LVEF

The earliest effort to automatically extract information from echocardiograms was by Chung (2005), who developed an information extraction system to identify 10 medical concepts and their associated values from narrative echocardiogram reports [9]. The system used UMLS through a MetaMap API. Evaluation of this approach using 403 manually annotated reports determined that the system possessed 78% recall and 99% precision.

In 2011, Garvin developed an NLP system for quality measurement using rules to capture EF in a project entitled ‘Automated Data Acquisition for Heart Failure’, undertaken by the US Department of Veterans Affairs. The investigators utilized a random sample of 765 echocardiograms from seven VA medical centers [10]. This system was called ‘Capture with UIMA of Needed Data using Regular Expressions for EF’ (CUIMANDREef). The training and test document sets were annotated by two to three experts, which categorized documents with similar characteristics into five distinct formats by manual inspection, considering outline, headers, and the location EF data for developing the algorithm. The system used rules to assess combinations of concepts in a document, and gave positive weight toward a score to classify outputs as being consistent with an EF >40% or an EF <40%. Section headings and locations in documents were used to resolve instances when multiple mentions of LVEF were present, giving precedence to the LVEF recorded in the conclusion section. Their system had accuracy of 99.8%. In a subsequent study, Mystre compared the regular expression-based approach of CUIMANDREef with a sequence tanning approach to detect references to EF. The latter was found to perform better, with an F1-measure of 95.0% (versus 89.1%) [11]. Additionally, Kim investigated domain adaptation approaches for EF extraction and reported an improvement in EF extraction accuracy [12].

Furthermore, Gobbel reported that the use of machine learning to assist annotators halved the time necessary for annotating HF-related concepts [13]. Recently, several studies have focused on extraction of EF [10, 14–18]. Notably, Patterson developed a regular expression-based NLP system using Java UIMA architecture for extracting LVEF along with 26 other cardiology concepts from echocardiograms, radiology notes, and clinical notes. The system was able to extract LVEF with a precision of 0.96-1.0.

Xie implemented an algorithm at Kaiser Permanente Medical Center that first segments ECHO reports into sections and sentences, and then searches for phrases suggested by a domain expert to extract numerical values or qualitative descriptions of EF in the vicinity of the phrase. The final stage involved determining if the EF was historical or negated using simple phrases. For validation, the algorithm output was compared with the annotation of a cardiologist for a random sample of 200 patients, and concluded that the system exhibited high accuracy, with sensitivity and precision values of 0.95 and 0.97, respectively [17].

The above studies to extract information from the echocardiogram have been carried out as a part of a broader area of research referred to as EHR-based phenotyping, as there is an increasing realization that the EHR data is not readily amenable for analysis, and information processing techniques are required to utilize the data for epidemiological research as well as for clinical decision support [19–22].

We have developed a regular expression-based NLP system to extract LVEF from echocardiogram reports. The intended use of our algorithm was to identify patients presenting heart failure with reduced ejection fraction (HFrEF) for driving a population-based therapeutic intervention program. We implemented the algorithm using Python scripts using Apache Spark cluster [23]. (see Appendix). The Python scripts and sample echocardiograms are available as open source (link to Github repository: https://github.com/waghsk/lvef-paper).

## Methods

This study was conducted at Partners Healthcare, Boston, and was approved by the institutional review board.

We queried the institutional research patient data registry (RPDR) to obtain a data set of 190,000 patients with coded heart failure, and further filtered this set to patients that were currently alive and had visited the health system in past two years [24]. Clinical notes for these patients were filtered to identify echocardiograms using codes for report-type that appear as meta-data fields. Then, we identified the codes for echocardiograms by manually examining random samples for each report type.

The study team included clinical experts with experience in manually extracting LVEF from the EHR chart. These experts also suggested that most notes had a table listing the ejection fraction or a paragraph with a heading for left ventricle that contained the EF. Accordingly, we designed a logic to extract LVEF by searching for 1) a tabular pattern, 2) a section for the left ventricle with numerical and range patterns, and 3) qualitative expressions in decreasing order of precedence. We took an iterative approach to implement the algorithm for LVEF extraction using regular expressions (see Fig. 2). The iterative approach is as follows: Implement code for a pattern, and execute on the entire corpus to generate a log, and then match results for each note. Based on the log output, the corpus was divided into a positive group that matched the pattern and a negative group that corresponded to ‘missing pattern’. A sample of the positive group was examined to ensure that the extracted value is accurate, and a random sample was then examined from the negative group to either extend the existing patterns or construct a new one. This process was continued until no inaccurate extractions existed in the positive group, and the accuracy of the negative group attained the desired threshold. A detailed distribution of the patterns is provided in Appendix 1.

After computing the LVEF value output for each echocardiogram, we identified latest LVEF value for each patient by sorting the echocardiograms by date and ignoring those from which EF could not be extracted. The extracted LVEF values were then divided into two categories: reduced EF (rEF) and not-reduced EF (-rEF) (see Table 2). Patients were correspondingly classified as having ‘Heart Failure with reduced Ejection Fraction’ (HFrEF) or not-HFrEF. The latter group is composed of patients presenting heart failure with mid-range or preserved ejection fraction’ (HFpEF or HFpEF).

**Table 2:** Distribution of pattern types for left ventricular ejection fraction mentions

EF Pattern Data Type	Count	%
Numerical	38,267	85
Range	4061	9
Qualitative	2768	6
No pattern matched	105	<1
Total	45,201	100

For validation, two sets of the most recent echocardiogram were manually annotated for the LVEF classes of LVEF<=40 and LVEF>40. The first set comprised 289 of the most recent echocardiogram reports classified as LVEF. The second set consisted of 50 of the most recent echocardiograms classified as LVEF>40. Then, manual annotations were compared with the system output to compute the confusion matrix and algorithm accuracy.

## Results

### Patients with coded heart failure diagnosis

We obtained all EHR data for patients that i) have ‘heart failure’ on their problem list, ii) have visited the hospital in the previous two years, and iii) are alive. Specifically, in ICD-10-CM, heart failure is coded as series i50, with 6 subcategories that further define the actual condition: combined systolic and diastolic heart failure, diastolic heart failure, left ventricular failure, systolic heart failure, other types of HF including (right heart failure), and unspecified heart failure. In addition to the ICD-10-CM codes for HF, we included any legacy ICD-9-CM, longitudinal medical record (LMR), and other institutional codes mapped into the ICD-10 codes.

‘Diagnoses \ Diseases of the circulatory system (i00-i99) \ Other forms of heart disease (i30-i52) \ Heart failure (i50)’.

### Echocardiogram detection

To identify echocardiograms from notes in the dataset, we searched notes for the phrase ‘ejection fraction’, and grouped notes by their meta-data type field, sorting them in order of decreasing frequency. Then, we examined a random sample of codes with over 1% frequency to determine if the codes corresponded to echocardiograms. There were 629 different codes. We extracted 3 samples for each of the codes, and manually examined the samples to delineate 37 codes corresponding to echocardiograms. The codes for echocardiograms were generally found to contain the prefix “ECH”. By filtering the notes using the echocardiogram codes and restricting the time period to the previous 2 years, we obtained a total of 46,634 echocardiograms belonging to 24,605 patients.

### Algorithm for extraction of LVEF from echocardiogram

**Fig. 1:**
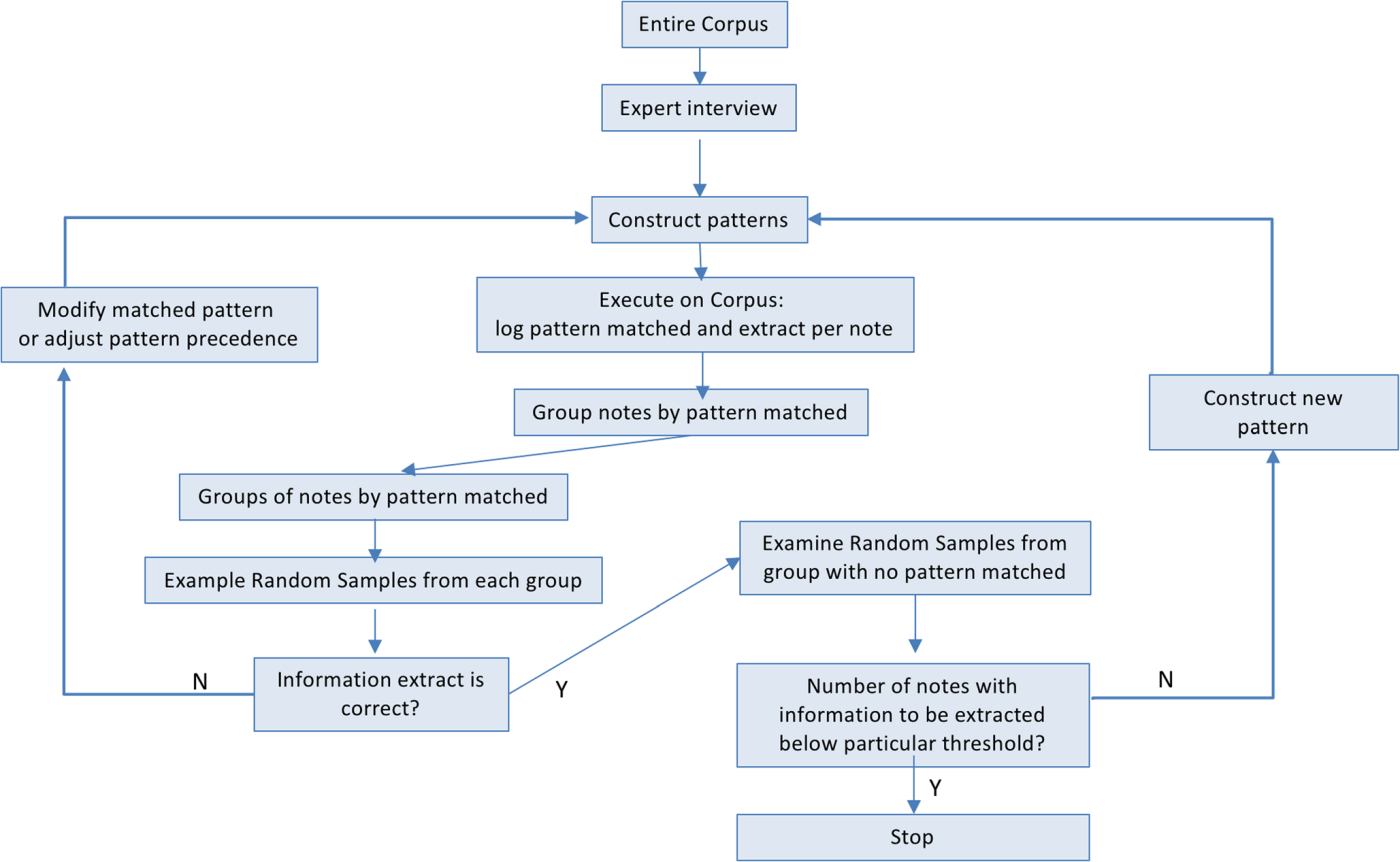
Steps to develop regular expression patterns for extracting LVEF

Section headings were identified in the notes by decomposing the corpus into lines and sorting out the most frequently occurring lines. These were then manually examined to construct a set of section headings. Using the section headings, we sliced each note in the corpus into sections and searched for the phrases ‘ejection fraction’, ‘lvef’, and ‘ef’ within sections with the heading ‘left ventricle’. We resorted to a regular expression-based approach, and analyzed the entire corpus using the approach summarized in Fig. 1.

**Fig. 2:**
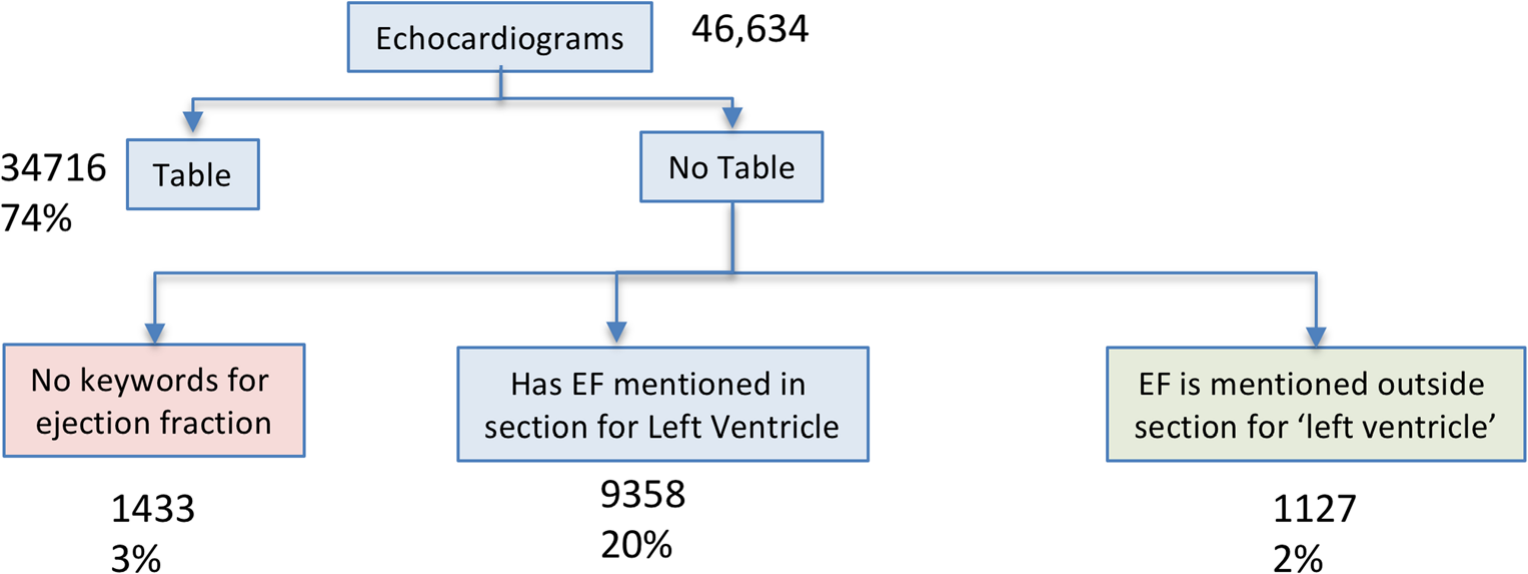
Distribution of high level echocardiograms categorization based on mentions of anchor terms for ejection fraction

The logic for parsing out EF from echocardiograms is described as follows. First, using meta-data, we ensured that the note to be parsed is an echocardiogram. Then, we searched for a tabular pattern with EF in the note. In the absence of a tabular pattern, we identified the section for left ventricle and searched for numerical and range patterns for EF. If none of these were located, we then searched for prose expressions. In the absence of such expressions, a log noted that no pattern was found (Table 1 and Fig. 2).

First, we developed regular expressions to extract EF from tables in echocardiograms, and then focused on the remaining echocardiograms to develop patterns for quantitative and qualitative descriptions of EF. Consequently, 4 major groups of patterns emerged: i) numerical, ii) range, iii) qualitative, and iv) no pattern (see Table 2).

**Fig. 3:**
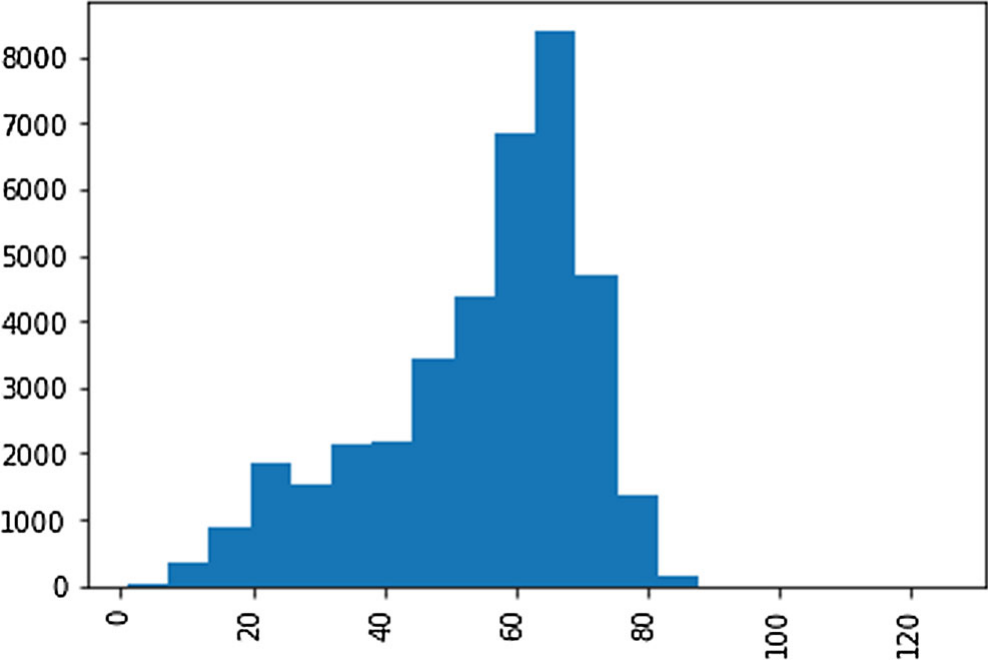
Distribution of numerical ejection fractions extracted from echocardiograms

**Fig. 4:**
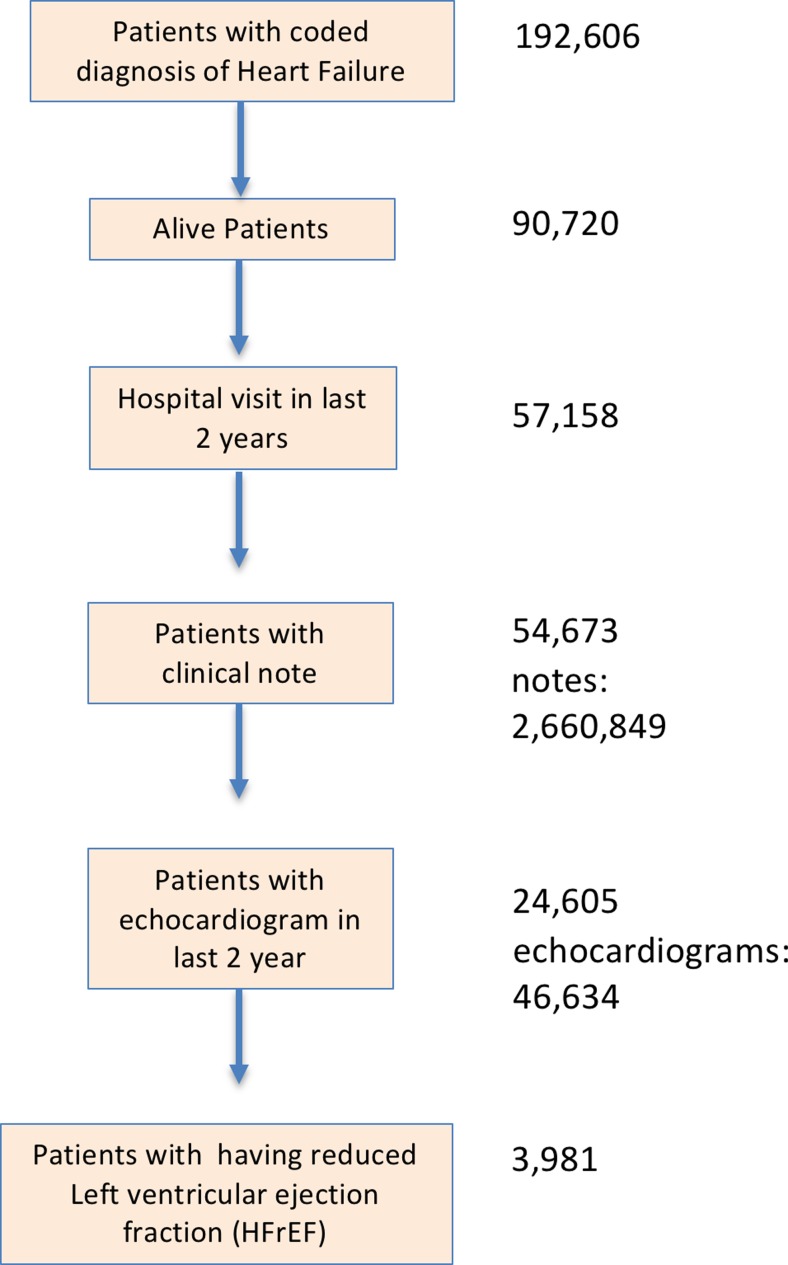
Consort diagram for the present study. Values to the right indicate the number of patients

**Fig. 5:**
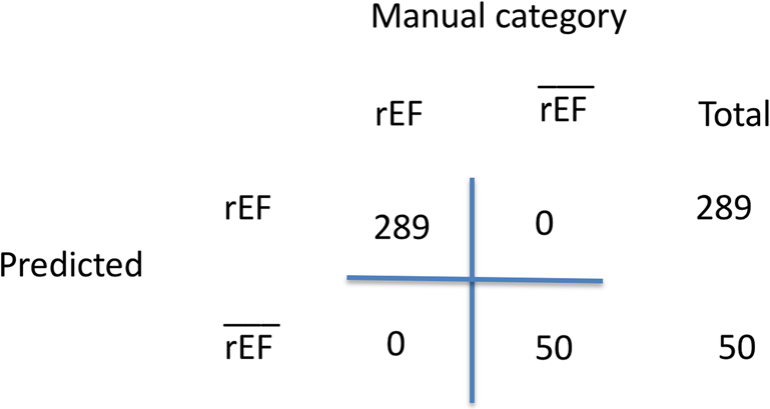
Confusion matrix for system validation

We classified the extracted EF ‘snippets’ to two classes, corresponding to EF ≤ 40 or EF > 40. First, the snippets with a single number were mapped. Next, the ranges (e.g. ‘40-50’ or ‘40 to 50’) were converted to an average. For qualitative expressions, we developed a lookup table to map the extracted text to the categories. The numerical values, ranges, and prose extracts had a distribution of 85, 9, and 6%, respectively. Less than 1% of notes with keywords for ejection fraction had no patterns matched for EF extraction. (Figs. 3, 4 and 5, Table 3)

**Table 3:** Distribution of numerical LVEF values for HF patient cohorts

Group Name	Range	Count	%
HFrEF	# < 40	6977	18
HFmEF	#40-50	5443	14
HFpEF	# > 50	25,847	68
	#total numerical	38,267	

### Manual validation to computer performance of the LVEF extraction algorithm

We compared the manual annotations with the algorithm output to compute the confusion matrix and accuracy of the algorithm. All instances of echocardiograms were correctly classified by the LVEF extraction algorithm, providing an accuracy of 1.0 (Fig. 5).

## Discussion

Our results demonstrate that an NLP algorithm using a regular expression-based approach was highly accurate in extracting LVEF from echocardiogram reports. This corroborates previous reports of high accuracy for LVEF extraction in other healthcare systems [16, 17, 25]. Use of regular expressions to extract information, though labor intensive, is known to yield high accuracy. Our approach described in Fig. 1 is helpful to systematically identify the patterns, while considering pattern prevalence to minimize the manual effort. An alternative approach is to automatically discover regular expressions from a training corpus, which is an active area of research [26–28].

We determined that 74% of extracted LVEF values were mentioned in tabular form, while this metric was 70% at Kaiser Permanente [17]. This difference is possibly due to the analysis of Xie being performed on a historical corpus, while the corpus of the present study was limited to the previous 2 years. We anticipate that improved integration of echocardiography software with EHR may have facilitated the granularity of EF reporting.

Many of previous approaches to LVEF extraction involved the creation of a gold standard, manually annotated by a domain expert that was then used to develop a text processing algorithm [25]. In contrast, we used an iterative approach, analyzing the entire corpus of echocardiograms to develop the EF extraction algorithm [17]. The developed algorithm was validated by manual inspection of a system output sample.

The disadvantage of developing a manually annotated corpus to guide system development is that it is labor intensive and can be expected to exhibit redundant instances of dominant patterns, as a random sample of instances will not be representative of the distribution of patterns to be extracted. For example, in the present study, a 70% random sample of instances for gold standard creation would have contained ejection fractions reported in the tabular form. However, these instances only corresponded to a small proportion of the regular expressions used in the system.

Knowing the prevalence of patterns in the text corpus is helpful for optimizing the effort invested in the implementation of processing logic for a certain pattern. Furthermore, such patterns can be escaped by logging an unknown pattern exception for the message. As shown in Fig. 1, we considered pattern prevalence distribution to guide the process of developing regular expressions for these patterns. The prevalence of the patterns for documenting EF is likely due to templates used during the data entry process. An important design feature of our algorithm was to give precedence to tabular mentions of EF, and only then consider section headers. This has been reported as being useful in previous studies [17, 25].

### Limitations

We restricted the scope of our analysis to the extraction of LVEF from echocardiograms. LVEFs can be found in echocardiograms as well as visit notes, wherein physicians summarize the findings of echocardiograms. As the study and associated interventions required objective evidence of low EF, we restricted the source of LVEF mentions to echocardiograms. A second limitation of our study is that a significant proportion of patients are expected to have echocardiograms from institutions outside the health system, which triggered referral to our hospitals for intervention. However, we restricted the analysis to echocardiograms conducted within the healthcare system to obviate the complexities of dealing with reporting variations outside the healthcare system.

## Conclusion

Our study demonstrates that a regular expression-based approach can accurately extract LVEF from echocardiograms. This algorithm can be utilized to delineate a cohort of HFrEF patients for implementing therapeutic interventions.

## Appendix 1

Python script to split the echocardiogram into sections and to identify the section header.

Python script to extract LVEF from echocardiogram from tabular format.

Distribution of patterns for extraction of LVEF from tabular form.

**Table:**

Pattern	Count
pat1	34,509
missing: no pattern matched	11,918
pat1_2	207
Total	46,634

Python script to extract LVEF from echocardiogram reports with a section for the left ventricle.

Distribution of patterns observed in echocardiogram reports with and without a section/paragraph for the left ventricle.

**Table:**

Pattern	In Section for the Left Ventricle	Outside Section for the Left Ventricle
pat2n	5482	489
pat8c	2450	212
pat4n	1159	258
pat3n	86	1
pat7c	77	16
pattern not found	52	53
pat5n	41	96
pat4_1c	11	2
Total	9358	1127

## Appendix 2

Python script to map numerical, range, and qualitative LVEF extracts to binary categories of reduced LVEF.
